# Area V5—a microcosm of the visual brain

**DOI:** 10.3389/fnint.2015.00021

**Published:** 2015-04-01

**Authors:** Semir Zeki

**Affiliations:** Wellcome Laboratory of Neurobiology, Cell and Developmental Biology, University College LondonLondon, UK

**Keywords:** V5, motion vision, dynamic parallelism, asynchronous visual processing, parallel processing, hierarchical processing, the Riddoch Syndrome

## Abstract

Area V5 of the visual brain, first identified anatomically in 1969 as a separate visual area, is critical for the perception of visual motion. As one of the most intensively studied parts of the visual brain, it has yielded many insights into how the visual brain operates. Among these are: the diversity of signals that determine the functional capacities of a visual area; the relationship between single cell activity in a specialized visual area and perception of, and preference for, attributes of a visual stimulus; the multiple asynchronous inputs into, and outputs from, an area as well as the multiple operations that it undertakes asynchronously; the relationship between activity at given, specialized, areas of the visual brain and conscious awareness; and the mechanisms used to “bind” signals from one area with those from another, with a different specialization, to give us our unitary perception of the visual world. Hence V5 is, in a sense, a microcosm of the visual world and its study gives important insights into how the whole visual brain is organized—anatomically, functionally and perceptually.

## Introduction

Most scientists who study the sensory cerebral cortex have an aim, often unacknowledged, which is to learn how activity in it contributes to our experience and knowledge of the world and therefore, implicitly, to how that activity is related to conscious experience. No visual cortical area exemplifies this better than Area V5, especially critical for the perception of visual motion. It is now one of the most intensively studied areas of the brain, rivalling even the primary visual cortex, area V1. Correspondingly, the number of papers published on it is enormous. I do not give an exhaustive review of the literature here, since many good summaries of its anatomical and physiological organization, and their relationship to motion perception, are available. I also restrict myself to a discussion of V5. There are other areas in the brain which may be involved with motion in other ways, including areas that V5 projects to directly (Howard et al., 1996; Sunaert et al., 1999; Gilaie-Dotan et al., 2013). I do not discuss these. Instead, I concentrate on those characteristics of V5 which, I believe, illustrate some general principles governing the organization and operations of the visual cortex at large and which bring us a little, but perhaps not much, closer to understanding the relationship between activity in a visual area and conscious knowledge. This makes of V5 a sort of window through which to look into the visual brain and its operations in general. The general principles I address in this essay are: the specialization of function in the visual brain, the diversity in the sources of signals contributing to the specialization of a visual area and enabling it to execute its functions, the cohabitation of asynchronous parallel and hierarchical processing in generating the functions of an area, the relationship of single cell responses to perceptual capacities, the restricted and degraded (phenomenal) conscious experience that is the correlate of activity in a visual area which is deprived of one of its parallel visual inputs, and the massively asynchronous operations of the visual brain. This, in turn, leads to a consideration of how the brain integrates signals related to different visual attributes to give us our unified visual experience.

V5 has also been a battleground of ideas, and served as a sort of passport for battling out larger issues, related mainly to functional specialization and visual consciousness. In this, too, V5 serves as a microcosm of the visual brain. It is a mistake to suppose that these issues, which have shaped our view of V5 and what its role is, are irrelevant to the overall picture of V5 that has emerged over the 45 years since its discovery or that they are not relevant to interpreting the functions of other visual areas, which is why I discuss them here.

## Historical Note

By the mid-1960s, area V1 was known to project to a wide region of cortex lying anterior to it, the “circumstriate cortical belt” (Kuypers et al., 1965). Area V5, also known as the middle temporal area (MT) or as V5/MT, was among the first visual areas in this belt to be delineated as a specific area, first in the macaque (Cragg, 1969; Zeki, 1969) and then, successively, in the owl monkey (Allman and Kaas, 1971) and human (Zeki et al., 1991) brains. It was also the first visual area outside V1 to which a specific function, that of visual motion, was assigned, in both the macaque (Dubner and Zeki, 1971; Zeki, 1974a) and human (Zeki et al., 1991) brains. That all the cells in it are indifferent to color, in an animal with good color vision, and commonly indifferent to their forms as well, suggested at once that color and form must be processed elsewhere, this being one of the key elements in ushering in the concept of functional specialization in the visual brain (Zeki, 1978a). The corresponding area, MT, in the owl monkey (from which the alternative name for V5 is derived) was initially charted only topographically through evoked potentials and was not characterized functionally until the 1980s (Zeki, 1980b; Baker et al., 1981). Although motion is also emphasized in it, its organization probably differs somewhat from that of the macaque. It has not played a significant role in revealing the cortical mechanisms underlying the perception of visual motion or of visual perception in general. I therefore consider area V5 in the macaque and human brains alone.

**Figure 1:**
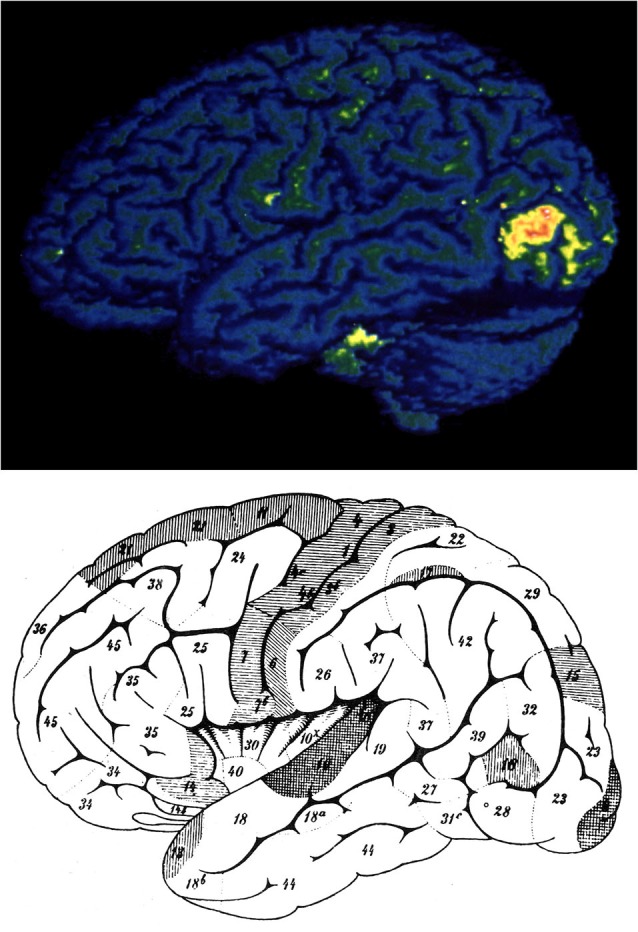
**The position of area V5 as revealed in human brain imaging experiments (above) and the position of Flechsig’s Feld 18 (below) For details see text**. From Zeki (1993).

**Figure 2:**
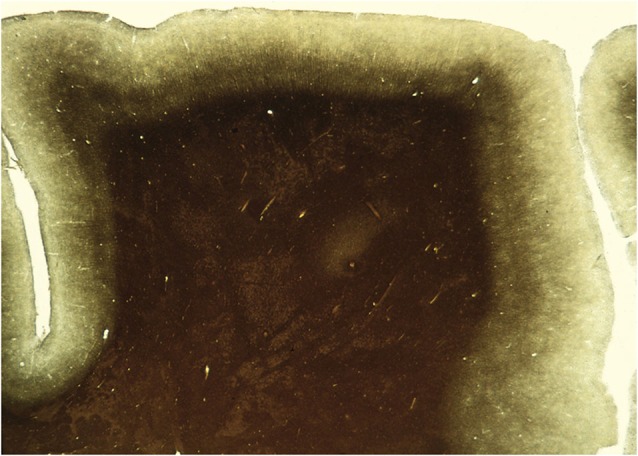
**A section through an area of the human brain corresponding to V5, stained by the Myelin method to reveal its rich myelination**. The boundaries of area V5 are clearly distinguishable.

A similar area in the cat brain had been described earlier by Clare and Bishop (1954), who considered their area to be an “association” area. The term “association” was not used as originally intended by Flechsig (1901), for whom “association cortex” was an anatomical term denoting areas that become myelinated after birth, unlike primary cortical areas, which are myelinated at birth. Soon, however, Flechsig (1905) began to think of his “association” areas as the *geistige* (mental)* Zentren* or *Cogitationzentren*, and the term “association” came to signify more than the sequence of myelination. Significantly, Flechsig demarcated one area in his “association” cortex, *Feld 16*, as being more fully myelinated at birth than surrounding cortex; it is in a position that is almost identical to the position of V5 in humans (Figures 1, 2). The earlier myelination of V5 has also been observed in other animals (Bourne and Rosa, 2006).

Clare and Bishop “inferred [their area] to comprise an association area relating optic and acoustic activity”, although no associational activity was studied there. Hubel and Wiesel (1969) recorded from its cells and found that the majority were directionally selective (DS). They nevertheless did not interpret the area to be specialized for visual motion, supposing instead that it continues the process of hierarchical analysis of visual form signals beyond V1. This led them to view the Clare-Bishop area as executing “the same processes” as earlier visual areas “but with different degrees of refinement”, leaving them “…with the puzzling prospect of an area for which we can…assign no obvious function” (Hubel and Wiesel, 1969) (see also Zeki, 1993 for a review).

Area V5 is also characterized by a heavy concentration of DS cells (Dubner and Zeki, 1971; Zeki, 1974a). But the interpretation we gave to its role was different. Instead of supposing that it continues the hierarchical process of analysing signals begun in V1, we assigned a specific function to it, characterizing it as an area specialized for visual motion. It is this specialization that, in one form or another, the great majority of studies in V5, in both macaques and humans, have concentrated on.

That visual motion may have a special representation in the brain was vaguely mooted in the early 1900s and easily ignored, partly because it had no credible supporting evidence and partly because it was stated in partial opposition to the prevailing views on area V1, not with respect to a specialized visual area outside it. In particular, Riddoch (1917) had examined patients blinded by lesions in V1, produced by gunshot wounds sustained during the Great War. He noted that the patients could perceive, consciously, visual motion in their “blind” fields. This led him to suggest that visual motion may have a special representation in the brain. Holmes (1918) was impatient with this view, as he was with all views that hinted at the presence of visual areas outside V1. Like others, he was quick to brush them aside. With Riddoch’s view on the representation of visual motion, this was easy. Riddoch had somewhat improbably, or so it seemed at the time, interpreted motion selectivity to be a manifestation of a specialization within V1 (then considered to be the sole visual area), not in cortex outside it. This made it difficult to understand how gunshot wounds in V1 could selectively spare one special visual mechanism alone, and therefore easy to dismiss the idea. General opinion at the time was in any case against such localization in the visual brain, summarized in the emphatic and dismissive statement by Holmes that, “…occipital lesions do not produce true dissociations of function with intact retinal sensibility” (Holmes, 1918). Riddoch’s evidence thus disappeared from the literature and resurfaced again after 1993 (see below). This is in spite of the fact that Poppelreuter (1923) had speculated that, “We have a plurality of different systems, which are affected in different ways and can also remain functional in different ways. i.e., the defect can show itself as specific for different systems”. Among these, he enumerated the motion, color, and form systems (see Zeki, 1993 for a review). However prescient his speculation in light of future developments, it remained mere speculation and had not the slightest influence.

## The Generation of a Specific Function—Visual Motion—in V5

It is generally agreed that one of the principle functions of V5 is to detect and signal the presence and direction of visual motion (Dubner and Zeki, 1971; Zeki, 1974a; Born and Bradley, 2005; Hock and Nichols, 2013) and that many of its cells are especially concerned with the overall, global, direction of motion of an object rather than with that of its component parts (Rust et al., 2006). The majority of studies, whether physiological or imaging, have concentrated on the capacity of V5 cells to register motion in the fronto-parallel plane and on their directional properties and speed preferences (Maunsell and Van Essen, 1983; Rodman and Albright, 1987; Priebe et al., 2003; Liu and Newsome, 2005). But V5 cells are also capable of signaling motion in depth, towards or away from the subject (Zeki, 1974b; Czuba et al., 2014; Sanada and DeAngelis, 2014) and indeed may be able to signal both fronto-parallel and 3-D motion (Huk, 2012). Its physiology is tailored, as well, to register many other aspects of depth in relation to motion (DeAngelis and Newsome, 1999; DeAngelis and Uka, 2003; Ponce et al., 2008; Nadler et al., 2013) and it probably has many other motion-related functions that remain to be charted.

Some try to downgrade the specialization of visual areas by emphasizing that depth detection and attentional mechanisms are also part of their functions (Roe et al., 2012). But these are general adscititious functions of all visual areas, including V5; they are critical for the functioning of all areas but do not define the core and specific role and functions of any. Any stimulus in the field of view will be at a certain distance, and therefore at a certain depth, from an observer. Correspondingly, all visual areas examined to date have been shown to be sensitive to depth (Bridge and Parker, 2007). This makes of depth detection a general function, even if each area processes depth in a way that is tailored to its physiology and function. Attentional mechanisms also form part of the repertoire of every visual area (Treue, 2003; McMains and Kastner, 2011) but may be utilized differently in different visual areas (Maunsell and Cook, 2002). They, too, do not in themselves define the function or special role of any visual area, even if the physiology underlying attentional mechanisms may differ between areas, depending on their primary specializations.

## The Emergence of Directionally Selective (DS) Cells in V5

**Figure 3:**
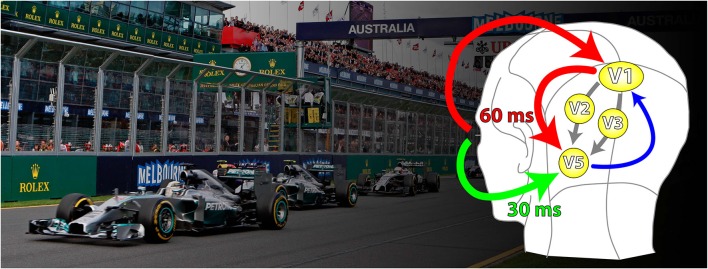
**A schematic representation of the parallel inputs to V5**. The classical input (shown in red) reaches V5 through V1 with latencies of about 60 ms. The V1-by-passing input (green) delivers its signals at latencies of about 30 ms. Its critical involvement in the detection of fast motion becomes evident in patients suffering from the Riddoch Syndrome, whose motion vision is a much impoverished but conscious capacity to detect the direction of motion of fast moving stimuli. There are further parallel (cortical) inputs to V5 in the classical pathways, from V2 and V3 (gray); the feedback from V5 to V1 is shown in blue.

DS cells in V5, with their specific receptive field characteristics, are generated by direct, hierarchically organized inputs from V1, by parallel inputs from V1, V2 and V3 and probably by intrinsic mechanisms within V5 itself. V5 also receives inputs from the the lateral geniculate nucleus (LGN) and the pulvinar but the latter do not carry directional signals (Berman and Wurtz, 2011; Figure 3).

### The Contribution of the Hierarchical Input from V1

It seems to be a general principle that, as one progresses through visual areas, the receptive fields of cells are enlarged, compared to the size of fields in V1. In V5, this enlargement is no doubt the consequence of the convergent anatomical input to it from V1 (and V2) (Zeki, 1971a), which endows V5 cells with larger receptive fields, capable of registering signals from wider regions of the visual field. This convergence also accounts partially for the motion properties of V5 cells, in particular their capacity to register the global motion of a stimulus instead of its component parts (Movshon et al., 1985; Simoncelli and Heeger, 1998; Priebe et al., 2003; Rust et al., 2006). The enlargement of fields is probably also the result of local connections that refine the substructure of the enlarged receptive fields of V5 cells (Majaj et al., 2007). Long range, clustered connections, which could be the source of such refinements, have been demonstrated anatomically within V5 (Ahmed et al., 2012). This is probably applicable to all such convergent connections within the visual brain, because convergence is unlikely to be the only source that specifies the somewhat complex sub-structure of these fields, without some local wiring, whether in V5 or elsewhere. As well, the “V1-bypassing” input to V5 almost certainly plays an as yet undetermined role in specifying the field structures of V5 cells.

The convergent outputs from V1 to V5 constitute an anatomical hierarchy, with the DS cells in V1 feeding into those of V5 to generate their properties in addition to their larger field sizes. This anatomical hierarchy is reflected in a physiological, functional, hierarchy, the general principle of which was first enunciated by Hubel and Wiesel (1962, 1965). They showed that orientation selective (OS) cells differ in the complexity of the stimuli to which they respond optimally, either within V1, or between V1 and further areas in prestriate cortex. This led them to view the hierarchical strategy as the organizing principle operating within the visual brain. One of the best demonstrations of functional hierarchy comes from studies in the visual motion system, and in particular in the functional relationship between DS cells in V1 and those in V5 (Movshon et al., 1985; Movshon and Newsome, 1996; Smith et al., 2005; Rust et al., 2006; Kiorpes et al., 2012). The basic picture that emerges is that the DS *and* OS cells of V1, located mainly in layer 4B and upper layer 6 (Lund et al., 1975; Ungerleider and Desimone, 1986; Shipp and Zeki, 1989a), detect the direction of motion of component parts of a stimulus, which might differ significantly from the overall motion pattern of the stimulus (Huk and Heeger, 2002). The latter appears to be detected by cells in V5. Not all V5 cells are of the pattern motion type; a significant percentage (25%) appears to be of the component motion type, suggesting that interactions within V5 itself may also contribute to the properties of pattern motion cells in it. Many questions still remain over the exact contribution that V1 (and V2) cells make to the elaboration of what Movshon and his colleagues have called pattern motion (see Born and Bradley, 2005) but, whatever the questions, it seems to be generally agreed that an emphasis on the true direction of motion of objects in all planes is a prominent feature of what V5 does. The model proposed by Movshon, Newsome and their colleagues is compelling; in broad terms, it is similar to the processes used in at least the color system, suggesting that it is a variant of a general strategy but tailored specifically for V5. In the color system, cells in V1 and V2 appear to be more potent in registering light of specific wavebands and are sensitive to changes in the wavelength composition of light reflected from their receptive fields (hence sensitive to components), while cells in V4 are capable of registering the color and hue, regardless of changes in wavelength composition (Zeki, 1980a, 1983b; Moutoussis and Zeki, 2002; Stoughton and Conway, 2008; Brouwer and Heeger, 2009, 2013), hence corresponding to the pattern motion in the motion system. Here again, the similarity is striking in that not all cells in V4 are true color cells; many still respond to specific wavelengths irrespective of the color (Zeki, 1983a,b), just as not all V5 cells are motion pattern detecting cells. This makes it plausible to suppose that the brain uses broadly similar strategies in building up different attributes of the visual world.

### The Contribution of the Parallel, Hierarchical Cortical Inputs from V2 and V3

V5 also receives parallel cortical inputs from other areas that V1 projects to, in particular from V2 and V3 (Zeki, 1971b; Shipp and Zeki, 1989b), the latter of which may be important in endowing V5 cells with the capacity to signal depth information (Adams and Zeki, 2001; Ponce et al., 2008). These parallel inputs have led to theoretical models which have tried to account, for example, for the generation of the illusory motion of illusory contours (Francis and Grossberg, 1996) but, in general, the roles of the V2 and V3 inputs to V5 have not been nearly as extensively studied as the one from V1. An important issue here, in the context of this article, is whether signals from V2 and V3 reach V5 at the same time as signals from V1 (see below).

### The Contribution of the Parallel Inputs to V5 that By-Pass V1: Dynamic Parallelism

While the hierarchical input to V5 from V1 accounts eloquently for many of the properties of V5 cells, and especially their capacity to register the true direction of motion of stimuli, it does not account for all. In particular, a ubiquitous finding is that, although DS cells in V5 have a range of speed preferences, most respond to relatively fast speeds, with a mean of 30° s^−1^, while the DS cells in V1 respond to slower speeds (<16° s^−1^) (Maunsell and Van Essen, 1983; Newsome et al., 1986; Rodman and Albright, 1987). The divide between cells that respond to fast motion and those that respond to slow motion emerges as one of the critical criteria for understanding one of the roles of V5 in motion. It is possible, and even likely, that preferences for higher speeds are mediated at least in part by a parallel input to V5 that by-passes V1, as well as by parallel cortical inputs to V5 from V2 and V3 (Ponce et al., 2011). It has long been known that prestriate visual areas, among them area V5, receive direct inputs from both the LGN and the inferior pulvinar (Cragg, 1969; Benevento and Rezak, 1976; Fries, 1981; Yukie and Iwai, 1981; ffytche et al., 1995a; Sincich et al., 2004; Leh et al., 2008; Shigihara and Zeki, 2013, 2014a). What has not been explored yet is how the “V1-bypassing” subcortical inputs to V5 co-operate with the parallel cortical input to it from V1 in sculpting the properties and receptive field structures of cells in V5 and other visual areas, especially since there may be a correspondence between directional preferences and speed tuning (Perrone and Krauzlis, 2008). The role of this “V1-bypassing” input to V5 is important, if only because faster speeds are the ones perceived (consciously) in subjects blinded by lesions in V1 which spare V5 (see under the Riddoch Syndrome).

Overall, we may derive two general principles from this section:

1. A general conclusion, possibly applicable to all visual areas besides V5, is that convergent, hierarchical, inputs from V1 endow cells in recipient areas with the capacity of responding to a stimulus in its entirety in terms of its specialization, rather than to its component “parts”; the latter are better registered in the earlier, feeding areas. Hence, specifying a function—in the case of V5, motion—involves at the same time an abstraction, since the specification in V5 is with respect to the direction of motion of stimuli composed of many parts, but the parts themselves, individually, are neither critical for the determination of the true, overall direction of motion of the stimulus nor, necessarily, of a V5 cell’s response to it.
2. That all “early” and “mid-tier” visual areas, as well as areas critical for the perception of houses and faces receive, like V5, two sets of parallel inputs (Shigihara and Zeki, 2013, 2014b): parallel inputs from more than one antecedent cortical visual area on the one hand, and parallel inputs from the cortex and the sub-cortex on the other (Figure 3). How the two sets of inputs determine and refine the characteristic physiology of an area remains largely unexplored.

## Diversity in the Source of Signals for Generating Specialized Functions in V5 and Other Visual Areas

The visual pathways that feed cortical visual areas have been divided into two broad classes, deriving their names from the magnocellular (M) and parvocellular (P) subdivisions of the LGN, with the koniocelluar (interlaminar) (K) a more recent addition (Hendry and Reid, 2000). Cells in the P layers have slow tonic responses and are commonly cone-opponent while those in the M layers are not and have faster, transient, responses. Both the M and P pathways receive input from all three cone types, while the K pathway receives short-wave vs. long- + middle-wave opponent cone inputs (Chatterjee and Callaway, 2002). When functional specialization was first demonstrated in visual cortex (Zeki, 1978b), no suggestion was made about the relationship of the specialized areas to the P and M pathways. When later Livingstone and Hubel (1988) addressed the issue, they proposed that the P and M dichotomy continues into the cortex, with the M pathway feeding into the motion system (and rendering it “color blind”) and the P pathway into the form and color systems (the K pathway had not been named as a contributor then); they further proposed equating these two subdivisions with the “what” and the “where” systems of Mishkin et al. (1983). My view was and remains significantly different; it supposes that, “an area performing a specialized higher function will tap any source of information that is useful” to execute its functions, be it the M, P (or K) components, even if one component constitutes the dominant input to one area and another component the dominant input to another (Zeki and Shipp, 1988). There is indeed good anatomical evidence that the M and K pathways are dominant in the input to V5, which can be inferred from the heavy bias in V5 in favor of fast motion. But there is good evidence that the M and P pathways, largely segregated up to the first cortical input stage, V1, are later intermixed to varying degrees (Lachica et al., 1992; Vidyasagar et al., 2002; Nassi and Callaway, 2009) and that, in addition to receiving input from the M and the K systems, V5 also receives a contribution from the P system, since blocking the P layers of the LGN can reduce the responses of V5 cells (Maunsell et al., 1990). This diversity in the source of signals is not unique to V5; it also occurs in V4 where, however, the P and M inputs are more equally weighted (Ferrera et al., 1994). The mixture of P, M and K signals in V1 and their wide distribution across prestriate cortex enables separate areas, whatever their specialization, to use signals from any source to undertake their tasks. Their capacity to do so would indeed be impoverished in the absence of a wide range of signals. An area such as V5 can detect motion generated from luminant and isoluminant stimuli, just an area such as V3B, for example, can detect form when generated from luminant and isoluminant stimuli (Zeki et al., 2003); this does not make of V3B an area that is specialized for motion (Van Oostende et al., 1997) any more than it makes of V5 an area that is important for color.

Reflecting the triple but M dominated input, the overwhelming majority of V5 cells are DS and care little about color, assuming them to care at all. However, V5 reacts to moving stimuli that differ in wavelength composition alone (isoluminant stimuli), not in luminance (ffytche et al., 1995b). Saito et al. (1989) found that the magnitude of response of V5 cells falls sharply when isoluminant stimuli (lacking all luminance contrasts), instead of luminous ones, are used to elicit responses from the DS cells there. Moreover, and critically, its cells do not have any preference for any particular chromatic combinations. Gegenfurtner et al. (1994) showed that the use of chromatically modulated stimuli reduced the responses of all V5 cells as the modulation neared the isoluminant plane. Significantly, they suggest that V5 cells may detect the motion of *fast* moving isoluminant stimuli (see below). Their conclusion, which I agree with, is emphatic, that, “although some MT neurons do give responses to isoluminant targets, they are unlikely to be the source of the chromatic motion signals revealed behaviorally”. In fact, perceived speed is very much reduced for isoluminant stimuli (Cavanagh et al., 1984) and the perception of slow motion may be mediated not only by V5 but by other visual areas as well, including V1, since its discrimination is relatively spared in patients with cerebral akinetopsia resulting from a lesion in V5 (see below).

There is a way in which every demonstration that a system uses signals that are not directly associated with its specialization turns into an assault on modularity and parallel processing, V5 being a good example of both. It may be useful to separate the behavioral demonstrations of interaction between color and motion from the responses of cells in visual areas in general, including V5, to moving isoluminant chromatic stimuli. Yet these are often not distinguished and lead—sometimes obliquely—to questioning functional specialization. Shapley and Hawken (2011) refer specifically to psychophysical experiments (of Wallach) to suggest that, “there is more interaction between color and motion than the modular view implies”. But nowhere does the modular view imply that there is no behavioral interaction between color and motion, nor does such interaction imply that the interaction must occur in V5 or that its cells are specialized for color as well as motion. There is any number of places where such interactions could occur. Shapley and Hawken (2011) see further support for their anti-modular stance by noting the anatomical connections between V4 and V5, which indicates to them “a departure from modularity of color and motion”. But such connections (which not all have observed Shipp and Zeki, 1995) may have other functions than that of endowing cells of V5 with color selectivity. By the logic of this argument, the connections to the intraparietal sulcus (IPS) from both V4 and V5 (Shipp and Zeki, 1995), should make of the cells there ones that respond to both color and motion. But the grouping of stimuli, or the formation of concepts, according to color or motion engages separate (and juxtaposed) subdivisions of the IPS (Zeki and Stutters, 2013; Cheadle and Zeki, 2014), suggesting that cells engaged in these activities are maintained separate even in a third area receiving inputs from V4 and V5. Gegenfurtner and Hawken (1996) found that 82% of V5 cells did not respond at or near isoluminance, leading them to the conclusion that V5 cells are not color opponent. They then make the claim that there is “no strict separation between color and motion *per se*”, thus falling into the fallacy of describing the nature of inputs rather than the specialization of outputs. In fact, color and motion are strictly separated at the level of V5, if not elsewhere, and this is evident in their own work. That would seem to constitute good evidence for parallel processing. The simple principle is that specialization depends upon the uses to which the inputs into an area are put, the nature of operations within the area, and the outputs from it.

In sum, the cells of V5 are not specialized for color to any significant extent, if at all, even if they, or at least some of them, receive input from the P system and from all three cone mechanisms. The papers of Saito et al. (1989) and Gegenfurtner et al. (1994) are not alone in reaching this conclusion. But, regrettably, use of the term “color” as a shorthand to signify cone-opponent inputs intrudes in a big and misleading way to suggest that color is a property of V5. This is sometimes misleadingly encapsulated in the title of papers which otherwise show nothing more than a cone-opponent input to V5 (Seidemann et al., 1999; Wandell et al., 1999). The trap is easy to fall into because isoluminant stimuli differ in color to human observers; it is nevertheless a mistake to suppose, or hint, from such demonstrations that V5 cells are selective for, or signal, color. After all, a great deal of successful effort has been expended on showing the relationship of single cell activity to motion perception but no one has yet succeeded, to my knowledge, in showing any relationship between V5 cells and the experience of color.

Cone-opponent signals can be used to define other features, besides motion. They can be used to detect forms, even when the ability to experience color is lost. For example, an achromatopsic patient, who could not recognize colors because of a cortical lesion, could nevertheless detect forms defined by differences in wavelength composition, though without being able to ascribe colors to them (Heywood et al., 1994). In fact, the ability of V5 to mediate the detection of motion using only a difference in wavelength between a stimulus and its background may be limited, even if it receives signals from the P and K systems, in addition to the M system. Two patients blinded by lesions in V1, who were able to discriminate motion, could nevertheless not do so when the difference between the moving spots and the background differed in wavelength composition alone (Alexander and Cowey, 2013).

Hence a diversity in the source of signals to an area does not, in itself, specify the functions of an area. It merely serves to broaden the range of signals which a visual area can use to undertake its specialized functions.

## The Relationship of Single Cell Activity in V5 to Perception

The search for the relationship between single cell activity in areas of the visual brain and perception of the attribute for which they are specialized has been a dominant feature in visual literature, and is especially well exemplified by V5. The evidence comes from three sources: brain imaging experiments, the study of the relationship of single cell activity to perceptual capacities, and clinical observations.

### Evidence from Imaging Studies

Perceived motion results in strong activation in V5 (Zeki et al., 1991; Watson et al., 1993), as inferred from changes in blood flow through the blood oxygen level dependent (BOLD) signal. V5 is also activated when subjects view images that imply motion (Kourtzi and Kanwisher, 2000; Fawcett et al., 2007; Kim and Blake, 2007). This raises the all-important question of “top-down” influences on the activity of V5, and also justifies, retrospectively, Flechsig’s use of the term *geistige Zentren* to describe his association cortex, though he did so intuitively rather than on the basis of such results. Moreover, the perception of fast motion that is entirely subjective and “illusory”, as in the fast motion in the rings of Isia Leviant’s static *Enigma*, correlates with activity in V5 (Zeki et al., 1993). It may be significant that V1 activity has not been observed in these studies, suggesting the possibility that it is dependent upon the “V1-bypassing” input to V5. Finally, the strength of activity in V5 is related parametrically to the declared preference for kinetic stimuli, the greater the preference, the more intense the activity there; with preferred kinetic stimuli, there is as well activity in field A1 of mOFC (Zeki and Stutters, 2012), suggesting a tight relationship between subjective preference for kinetic configurations and strength of activity in both areas. This is actually somewhat remarkable. It suggests that a sensory area may have some role in mediating subjective preferences. This complements earlier studies which show that microstimulation of cells in V5 can bias a monkey’s judgment of direction of motion (see below) and therefore that a visual area may have functions beyond that of processing sensory signals.

Even in spite of this impressive list, the relationship between V5 activity (as inferred from blood flow changes) and perceived (or unperceived) motion is not straightforward. In dichoptic experiments, two opposite directions of motion presented to the two eyes elicit a weak percept of motion but strong activity in V5. By contrast, the presentation of identical directions to the two eyes results in a strong perception of motion but weaker activity in V5 (Moutoussis and Zeki, 2008), presumably because more DS cells are activated when two opposite directions, as opposed to only one, are presented. Moreover, not all activity in V5 reaches conscious awareness since motion information in a peripheral location of the field of view, though imperceptible to humans, can modulate activity in V5 (Moutoussis and Zeki, 2006). These results suggest that a direct relationship between activity of V5 cells and (conscious) perception may be the privilege of sub-populations of cells in V5.

### Evidence from Single Cell Studies and Micro-Stimulation

Such sub-populations may have responses that are closely linked to the perception of motion and to decisions dictated by the direction of motion of a stimulus, as established in a series of studies by Movshon et al. which have explored more rigourously than for any other visual area the relationship between single cell activity, perception and judgments based on it. In combined psychophysical-physiological experiments, they have shown a close relationship between single DS cell responses and the directional strength (degree of correlation or coherence between moving dots) of moving signals (Britten et al., 1993), although some cells appear to have outperformed the animal while others did not do as well. A similar result has been obtained for overall activity in human V5 with imaging experiments (Rees et al., 2000), although the similarity may not extend over prolonged time periods (Kayser et al., 2010). Especially interesting is the observation that micro-stimulation of V5 cells with particular directional preferences can influence a monkey’s choice in discriminating the direction of motion (Salzman et al., 1992), just as stimulation of disparity tuned cells there biases their perceptual judgment of depth (DeAngelis et al., 1998). As interesting, in view of V5’s critical role in the perception of high speeds, even in the absence of V1, is the observation of a good match between psychophysical detection of high speeds and activity in V5 (but not in V1); by contrast, low speed detection correlates well with the activity of directional cells in both V1 and V5 (Newsome et al., 1986). This supports evidence which shows that signals from both fast and slow moving stimuli reach V5, but at different latencies and through different routes, fast signals reaching V5 predominantly through the “V1-bypassing” pathway and having a significant temporal advantage over slow ones, which reach V5 predominantly through V1 (ffytche et al., 1995a; Buchner et al., 1997; Gaglianese et al., 2012).

A tight relationship between psychometric functions and cell responses is also a feature in other prestriate visual areas (Kusunoki et al., 2006). But the evidence has been taken much further in V5. In particular, micro-stimulation of DS cells in V5 not only affects a monkey’s discrimination performance but also its decision to move its eyes in the corresponding direction (Britten et al., 2009). An important suggestion from these studies is that psychophysical decisions pertaining to motion may be mediated by a relatively small number of neurons (100 or multiples thereof) (Britten et al., 1992; Shadlen et al., 1996). Such a conclusion is relevant to the multiple parallel, but asynchronous, operations that an area may undertake (see below).

Collectively, these impressive findings establish the all-important link between single cell activity and perception as well as the link between that activity and judgment and decision making. But since this may be the privilege only of sub-populations of cells in V5, it raises the question of the relationship between such privileged cells and others, whose activity does not have an explicit perceptual correlate. It also raises the question of parallel, and possibly asynchronous, operations undertaken by different groups of cells within V5, because the privilege may not be absolute but bestowed on sub-populations according to the task undertaken (see below). Before addressing these, I review evidence to show (a) that the speed of delivery of signals to V5 may in part determine the relationship between activity in V5 and behavior; and (b) that a much diminished visual input to V5 can nevertheless mediate the perception of fast directional motion, implying that the activity of a relatively small number of cells in V5 may be sufficient for the conscious perception of directional motion.

## Asynchrony of Inputs to V5 and Its Consequences

### The Asynchronous Delivery of Signals to V5 by the Parallel Pathways

The pathways relaying signals to V5 do not deliver them synchronously. A somewhat counter-intuitive result was obtained from experiments which tried to disable the perception of motion by applying transcranial magnetic stimulation (TMS) to V1 and V5 (Beckers and Zeki, 1995). The results suggested that signals from fast moving stimuli reach V5 before reaching V1 and that signals from slow-moving stimuli, which reach V5 through V1, do so later. Recording experiments using EEG coupled to MEG, found that signals from stimuli moving at speeds of over 22° s^−1^ reach V5 directly at about 30 ms after stimulus onset while those at speeds lower than 6° s^−1^ are routed through V1, to reach V5 at about 60 ms after onset (ffytche et al., 1995a; Buchner et al., 1997; Schoenfeld et al., 2002; Gaglianese et al., 2012) (but see also Rao et al., 2001) and that this fast delivery of signals to V5 is preserved in a patient with a V1 lesion (ffytche et al., 1996). Thus, whether motion signals access V5 or V1 first depends on the speed of the moving stimulus, which regulates the sequence of arrival of signals according to the principle of *dynamic parallelism* (ffytche et al., 1995a) (see Figure 3). This suggests that a heavy proportion of V5 cells must receive input from the “V1-bypassing” pathway since most respond optimally to speeds in excess of 10° s^−1^, peaking at 32° s^−1^, whether in the anesthetized (Maunsell and Van Essen, 1983) or behaving (Mikami et al., 1986a,b) monkey. It is also consistent with evidence from subjects with lesions in V1 and in V5. If V5 is spared in subjects with V1 lesions, they are capable of discriminating consciously the direction of fast but not slow moving stimuli (see the Riddoch Syndrome below). If the damage involves V5 but spares V1, the patient is only capable of discriminating slow motion (Zihl et al., 1991; Shipp et al., 1994) (see under Akinetopsia below). The heavy myelination of V5 (see Figure 2), which implies that the input to it, as well as the interhemispheric connections between V5 in the two hemispheres, consists of large myelinated fibers, is no doubt, at least partially, the basis of this rapid transmission of signals to it.

To this asynchronous delivery of signals to V5 from the two pathways must be added the inputs which reach V5 from areas such as V2 and V3, which likely arrive in V5 after the signals from V1 and from the LGN are delivered to it. This means that V5 must handle a variety of inputs reaching it at different times.

### Asynchronous Operations Within V5

Given that the parallel inputs to V5, either those from the cortex (V1, V2, V3), or those from the cortex and the subcortex, deliver their signals to it asynchronously, it becomes interesting to address the question of whether there are multiple processing systems within V5, undertaking their tasks asynchronously, depending upon the characteristics of the stimulus. Smith et al. (2005) have found that responses of cells that signal component motion in V5 start about 6 ms earlier than that of cells that respond to pattern motion and that it takes about 50–75 ms for pattern motion cells to build up their selective profile, suggesting a temporal hierarchy. 75 ms is considerably longer than the shortest latency activation recorded in V5 with fast moving stimuli (ffytche et al., 1995a; Buchner et al., 1997). This raises two questions: can the activity produced by the fast input to V5 become perceptually explicit (that is, require no further processing) without support of the input from V1 and how is this “V1-bypassing” input integrated into the temporally hierarchical elaboration of pattern motion cells in V5 in the normal brain? It is unlikely that such integration occurs in Riddoch Syndrome patients, who are blinded by lesions in V1, thus depriving V5 of a principal source of its cortical input; such patients are able to perceive the direction of fast motion without at the same time being able to perceive any of the details of the moving stimuli. It does not follow from this that integration between fast and slow signals does not occur in normal V5, but the dynamics of this have yet to be addressed.

The time-based activation studies mentioned above imply, theoretically at least, that the activity of cells detecting fast motion and driven by the “V1-bypassing” input may become perceptually explicit before the activity of cells that signal pattern motion, which are driven by V1 inputs. This alone makes it possible, and even likely, that V5 processes separate motion-related signals asynchronously. It is also possible that other stimulus-related features, for example motion in depth, are also processed asynchronously with respect to motion in the fronto-parallel plane. This would make of V5 an area that processes several distinct, but motion-related, signals separately, in parallel, and asynchronously. Indeed, V5 may have sub-components that are specialized for specific motion features such as optic flow (Morrone et al., 2000), or a clustering (even if a relatively weak one) according to speed of motion (Liu and Newsome, 2003, 2005), implying that different groupings in V5 may process signals relatively independently from each other.

Cells in V5 can register more than one characteristic related to their specialization. They can, for example, be capable of signaling motion in the fronto-parallel plane as well as in depth. Disparity and non-disparity selective sites within V5 may differ (DeAngelis and Newsome, 2004). The effects on directional motion judgment in monkeys appear to be dependent more on micro-stimulation of non-disparity selective sites in V5 (i.e., the effects are obtained by micro-stimulation of directional columns alone). Such judgments can nevertheless also be influenced by micro-stimulation of V5 sites with strong disparity preferences, implying that whether disparity information is utilized or not depends upon the strategy used by the monkey to perform its task (DeAngelis and Newsome, 2004). This raises the very complex issue of whether the task-dependent presence or absence of this “cross-talk” follows different time-courses in dictating judgment. In his review of V5 cells that may encode both fronto-parallel motion and motion in depth, Huk (2012) writes that circuits can “…carry multiple signals simultaneously, or at least have the capability to carry different signals at different times or under different conditions”, raising the question of whether the decoding of signals is solely under cognitive control. If other factors come into play, then it becomes interesting to enquire whether decoding itself is asynchronous, depending upon the factors that come into play.

It is, in sum, unappealing to suppose that V5 waits for all the inputs to reach it before starting to process them, for that would suppose that the initiation of processing depends upon the operation of a central timing mechanism and no such “clock” has ever been identified. More likely, the multiple parallel processings that V5 can evidently undertake are also asynchronous with respect to one another.

It is unlikely that V5 is unique in this; more likely other nodes in the visual pathways also process a variety of signals related to their specialization but used for different ends in relation to that specialization. Even if signals reach different visual areas in parallel and synchronously, it does not follow that they process their signals synchronously. In the visual form systems, signals apparently reach V1 and the prestriate areas (V2 and V3, or the visual areas critical for the perception of faces and houses) within the same time frame, as is shown by the parallel inputs to V1 and prestriate areas by perceptually simple and complex form stimuli (Shigihara and Zeki, 2013, 2014b). But, just like V5, prestriate areas such as the ones enumerated above also handle signals that reach them through V1, which may be asynchronous with respect to the signals reaching them directly from the LGN and pulvinar, pointing to possible asynchronous operations within these areas. The relationship of synchronous and asynchronous parallel inputs to synchronous and asynchronous operations within individual areas has not been tested and merits future study.

### Asynchronous Outputs from, and Return Inputs to, V5

Such putative asynchronous operations within V5 and between V5 and other areas are pointers to other asynchronies in the visual brain, which include asynchronous outputs and asynchronous top-down operations. V5 has outward, feed-forward projections to other areas, including to areas surrounding it (which, together with V5, constitute the V5 complex) as well as to parietal cortex (Shipp and Zeki, 1995) and it is natural to assume that the results of activity in the former are communicated to the latter. Whether signals are continuously communicated in these pathways, even before the processing reaches a perceptual endpoint (as defined above), has not been determined, or even addressed. Unless there is some clock within V5 that dictates that the output from it is activated only when all the operations that it is involved in are complete, it becomes reasonable to suppose that there are separate, and asynchronous, outputs from V5 and other visual areas. There is evidence to suggest that activity in different groups of cells within V5 reach perceptual end-points at different times (Lo and Zeki, 2014), just as activity in different visual areas reach perceptual endpoints asynchronously (Moutoussis and Zeki, 1997b; Arnold et al., 2001). Moreover, recent experiments on color and form suggest strongly that the outputs from two distinct visual areas (as opposed to the same area) are asynchronous (Rangelov and Zeki, 2014).

The asynchronous operations and their consequences, described above, may in fact be more general because the functional specialization evident in the organization of the visual areas of prestriate cortex is also evident at higher levels of organization. For example, separate but contiguous divisions of IPS, receiving input from V4 and V5, respectively, are engaged in grouping or the formation of concepts according to color and motion, respectively (Zeki and Stutters, 2013; Cheadle and Zeki, 2014). But there are, of course, also backward inputs to the visual areas from higher centers. Are these reverse inputs sending signals continuously or after some determined but as yet unspecified and unknown end-point? It is hard to believe that asynchronous processing does not somehow regulate the timing of the return input to the prestriate visual areas which themselves process signals asynchronously. The prediction error system (Mumford, 1992; Rao and Ballard, 1999; Friston and Kiebel, 2009) provides a good example. This feed-back system is thought to operate widely on perceptual systems that are themselves asynchronous in their operation, implying that the results of action of different “top-down” systems are not applied simultaneously, since that would involve waiting for all the processing systems to complete their tasks, and there does not appear to be a “waiting” system. More likely, the massive error prediction system must itself act asynchronously.

## The Riddoch Syndrome

If V5 undertakes several operations in parallel and asynchronously, it is conceivable that each one of these may be demonstrable in isolation. A relatively simple way of addressing this is to assess behavioral capacities in terms of motion perception when V5 is isolated from V1, which should give strong hints as to what perceptual capacities a subcortical input to V5 can confer. At issue is an important question: Can activity in V5 that is disconnected from V1, and hence does not receive input from, or return outputs to, V1, have a conscious correlate? We recall, that the direct, “V1-bypassing”, pathway that reaches V5 appears to deliver preferentially signals from fast moving stimuli and is therefore in dynamic parallelism with the parallel pathway through V1, which delivers signals from more slowly moving stimuli to V5.

In the first study of its kind Rodman et al. (1989) showed that, after disconnection from V1, a majority of cells in V5 remained visually responsive (though with diminished vigour) and maintained their receptive field sizes and characteristic DS properties. As well, the overall topography of V5 was not significantly modified, thus suggesting that the “V1-by-passing” pathway to V5 must be organized topographically and contribute significantly to the structuring of the receptive fields of its cells. In humans, this study was presaged by an earlier study (Poppel et al., 1973) showing that patients blinded by lesions in V1 can respond non-verbally (by eye-movements) to stimuli appearing in their blind fields, leading the authors to raise the possibility that, “there may remain in the visual cortex a representation of the visual field which… is not revealed by ordinary perimetry”.

V5 is not unique in this; evidence shows that responses in other visual areas are also not abolished by de-afferentation from V1. Cells in V2 and V3 are reactive to the appropriate visual stimuli in the absence of V1, though with much diminished strength (Schmid et al., 2009). But, in charting the contribution of the direct “V1-bypassing” pathway, V5 has so far been a more productive playground than other visual areas. What has emerged is that the directional properties of cells in V5 is only “minimally” conferred on them by an input from pulvinar; rather it is the reverse input, from V5 to pulvinar, that confers on the latter the property of directional selectivity (Berman and Wurtz, 2011). This suggests that it is the organization of V5 itself that confers directional properties to the input from the pulvinar, emphasizing yet again the importance of distinguishing the input from the operations undertaken by an area.

Hence V5 can build up directional selectivity from the “V1-bypassing” pathway. How this directional selectivity is modified, or refined, by the one conferred on V5 by the input to it from V1 remains to be clarified. But, while showing that a “V1-bypassing” input to V5 can sustain activity in it, in response to visual stimulation, these animal studies also supposed, either explicitly (Bullier et al., 1994) or implicitly (Rodman et al., 1989) that V5 activity due to such an input does not reach conscious experience. Human studies were to show otherwise.

Conscious experience is naturally easier to study in humans and a good test of whether activity in a V5 de-afferented from V1 can acquire a conscious correlate lies in studying the visual capacities of patients in whom V1 is damaged. This has been done many times and involved in particular a patient, GY, blinded unilaterally by a lesion to V1 which spared V5, sustained in childhood. Over a long period, the assumption was that GY was able to discriminate visual stimuli presented to his blind field, without being aware of the stimuli themselves. This led to the supposition that passage of visual signals through V1 (Weiskrantz, 1986) or a return input to it from V5 (Lamme, 2001), or both, are essential for conferring a conscious correlate on what is processed in V5. This syndrome, apparently consisting of a perfect capacity to discriminate stimuli presented to the blind field in the total absence of awareness of the stimuli, was named Blindsight. Far reaching conclusions, even philosophical implications, were read into it, including the supposition that the perfect but unconscious discriminative capacities of such cortically blinded patients can be accounted for by subcortical mechanisms involving structures such as the superior colliculus (Weiskrantz et al., 1974). It was supposed, in brief, that “conscious vision is not possible without V1” (Stoerig and Cowey, 1995). It is a phenomenon long subscribed to by neurobiologists, with varying degrees of enthusiasm. I myself subscribed to it (Zeki, 1993). By implication, it was also supposed that the direct, “V1-bypassing” input to V5 from the LGN is insufficient to sustain conscious activity (Weiskrantz, 1986).

A turning point came with a further study of GY (Barbur et al., 1993) which raised doubts about the syndrome of Blindsight. GY turned out to be aware of the directional motion of stimuli—usually, and significantly, high contrast, fast moving ones—which he could discriminate, thus suggesting that there could be conscious vision without V1 (Barbur et al., 1993), even though GY’s visual experience of fast motion is much degraded, compared to that of subjects with an intact V1. On the other hand, he was not able to discriminate stimuli in slow motion which he was also unaware of. Others have since reached similar conclusions (Ceccaldi et al., 1992; Benson et al., 1998; Zeki and ffytche, 1998; Morland et al., 1999; Stoerig and Barth, 2001; Overgaard et al., 2008; Sahraie et al., 2010). Moreover, this conscious experience of fast visual motion by GY correlates with activity in V5 (see also Zeki and ffytche, 1998), hence suggesting that activity in V5 which has no input from V1 and no feed-back to it, can sustain a crude but conscious experience of fast, high contrast, motion, not only in GY but in other similar patients as well (ffytche and Zeki, 2011).

Because the motion vision of such patients is very impoverished, one should not expect that a patient blinded by lesions to V1 will be able to detect every single configuration of fast motion. Noting that patients blinded by lesions in V1 are unable to discriminate the direction of motion of random dot kinematograms moving at 20° s^−1^1, Azzopardi and Cowey (2001) write that “motion processing is severely impaired after striate cortex lesions. If this is the case, then there are no grounds for inferring that there is an alternative route to the extrastriate visual cortex that supports motion perception adequately”. Well yes, but it does support it inadequately nevertheless. Important though such demonstrations are, it is equally important to learn what perceptual experiences V5 is capable of mediating, especially in relation to the minimum conditions required for conscious experience.

The 1993 study of Barbur et al. (1993) led the proponents of Blindsight to re-label Blindsight into *Type 1* and *Type 2* (Weiskrantz et al., 1995), the former corresponding to its original definition and the latter to the discovery that GY and other patients like him can be conscious of stimuli presented to their blind fields. In fact, a careful reading of the Blindsight literature before 1993 shows that conscious awareness in such patients is common but was not taken account of, presumably because it is so crude and impoverished (Zeki and ffytche, 1998). At any rate, the re-labeling of Blindsight into two types, of which one is accompanied by conscious awareness, constitutes an acknowledgment that such subjects can be aware of some types of visual stimuli presented to their blind field. But the acknowledgment came with a price; the goal posts shifted endlessly (ffytche and Zeki, 2011) and a premium was now placed on deciding whether the acknowledged awareness was visual or not, some kind of “feeling” produced specifically by visual stimuli. This eristic diversion is one that nevertheless acknowledges the presence of awareness (see discussions by Foley, 2015; Kentridge, 2015; Macpherson, 2015; Overgaard and Mogensen, 2015). This should have of course been acknowledged long before the study of Barbur et al. (1993). George Riddoch had described how his patients, blinded by lesions in V1, could discriminate *consciously* the presence of motion in their blind fields. He wrote of “the frequency with which [such patients]…were immediately conscious of “something” moving”, adding that they were “…quite sure that neither shape nor colour can be attributed [to the movement]”; he emphasized that, “The patients have great difficulty in describing the nature of the movement that they see; it is so vague and shadowy” (Riddoch, 1917). In spite of the many pages written on the subject, I doubt that anyone has produced a better summary. We have reviewed this topic and its history elsewhere (Zeki and ffytche, 1998; ffytche and Zeki, 2011), and have referred to this syndrome as the Riddoch Syndrome, in honor of George Riddoch. He was the first to describe it, though without naming it. He was never credited for it.

There is now a general consensus that fast moving, high contrast stimuli are the ones which such subjects are aware of. In 1998, we wrote that “there is a correlation between GY’s capacity to discriminate and his awareness. The correlation… is not absolute (owing) to the fluctuating level of both his visual awareness and his discrimination performance. His level of awareness under particular stimulus conditions can vary between sessions” (Zeki and ffytche, 1998) (see Figure 5). The proponents of Blindsight have now acknowledged this, by stating that, “It is more likely that awareness would fall on a continuum rather than a discrete scale” (Sahraie et al., 2010). Arguably, this renders the distinction between Type 1 and Type Blindsight redundant.

**Figure 4:**
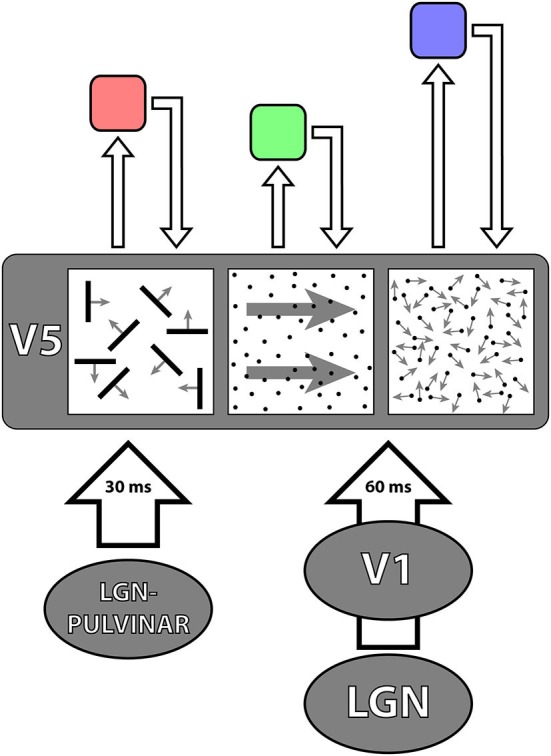
**A simplified diagrammatic representation to illustrate the principle of asynchrony**. The two parallel inputs to V5, from V1 and directly from the LGN—pulvinar, deliver signals to it asynchronously, in 30 ms from onset for fast motion and 60 ms from onset for slow motion. It is likely that V5 itself undertakes its multiple operations asynchronously, from which it follows that the outputs from, and the return inputs to, it from higher areas (of which three are shown above in pink, green and violet) that regulate its activity, must also be asynchronous. The latencies with which signals are delivered from V5 to higher areas are not known and nor are the latencies with which signals are delivered back to V5 from higher areas. The length of the arrows is therefore merely to indicate that they must be asynchronous.

**Figure 5:**
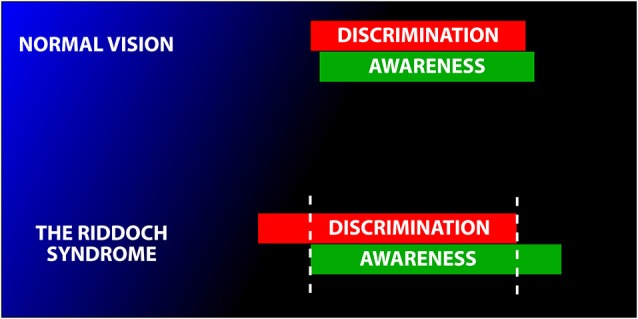
**Schematic diagram to illustrate the variable relationship between awareness and discrimination**. In normal subjects, the two are tightly coupled (above). In Riddoch Syndrome patients the two are partially dissociated. This leads to the condition in which Riddoch Syndrome patients are usually, but not always, able to discriminate what they are aware of (with fast moving stimuli) and, conversely are usually unable to discriminate the direction of motion of stimuli of which they are unaware. Modified from Zeki and ffytche (1998).

This is not to say that the debate about conscious awareness in such blind patients, triggered by showing that patients blinded by V1 can have a conscious experience of the stimuli presented in their blind fields, did not yield a mass of interesting results which are worthy of further study. But the central point I am making here is that activity at a node such as V5, disconnected from V1 and hence neither receiving inputs from it nor returning outputs to it, can have a crude conscious correlate. This naturally calls into question the supposition that, in order for activity in V5 to acquire a conscious correlate, “pre-processing” (whatever that may mean physiologically) by V1, and referral of signals back to it, are mandatory (Lamme, 2001).

In an article entitled *The Blindsight Saga*, Cowey (2010) wrote of Blindsight as being highly controversial. What makes it so are not the results of experiments and observations, which consistently have shown that Blindsight patients do have some crude awareness of stimuli that they discriminate, a critical fact that was consistently ignored in the pre-1993 literature on Blindsight. Rather, it is the implication that activity in a visual area, produced by a visual input that by-passes V1, cannot have any conscious correlate without the active participation of V1, either through feed-forward or feed-back connections. This condemned activity in the prestriate cortex as one that is “unconscious” without the participation of V1. Judging by the mass of papers addressing this topic since 1993, this is evidently not true for V5. It is likely to be untrue for other specialized prestriate visual areas as well, as the relatively small number of studies we have on the subject show. For example, patients blinded by lesions in V1 can have a rudimentary experience of colored stimuli if they are large (Brent et al., 1994). Future studies may reveal the same degree of autonomy for other visual areas as well.

Salomon Henschen, who was the first to identify human V1 and equate it with the striate cortex, was extremely hostile to the notion of any visual areas outside his “optic retina” (V1). Like Gordon Holmes, he cared nothing for clinical evidence that purported to show a color center outside V1; they both dismissed, very effectively, the evidence presented by a handful of neurologists to that effect (Zeki, 1990). To Henschen, the existence of visual centers outside V1 went against reason for, if true, then with V1 destroyed and the remaining cortex intact, “a patient would have to be absolutely blind and yet be able to see colors, which makes no sense” (Henschen, 1910). What made no sense over a century ago makes sense now for motion and color. It may yet turn out to make sense for other attributes as well.

### Akinetopsia and Motion Vision After Damage to V5

Riddoch’s evidence that (conscious) motion vision can be selectively preserved in subjects who were apparently blinded by lesions to V1 was universally but unjustly ignored. The early but nebulous clinical evidence for the syndrome of akinetopsia (motion blindness) (Zeki, 1991) following cortical lesions was also universally ignored, but this time with more reason (Zeki, 1993). It was clinical observations, derived principally from the work of Zihl and his colleagues from the 1980s onwards, which made the syndrome of akinetopsia acceptable. Once again, the divide between fast and slow motion appears as critical for separating the motion vision of akinetopsic patients from those suffering from the Riddoch Syndrome.

The best evidence that V5 lesions in humans compromise severely the perception of fast, but not slow, motion comes from the study of patient LM (Zihl et al., 1983; Hess et al., 1989; Zihl et al., 1991; Shipp et al., 1994). She had sustained bilateral damage to prestriate cortex, including the territory of V5, but considerably more extensive (Shipp et al., 1994), resulting in a long-lasting akinetopsia. The evidence from LM is, in brief, consistent with evidence that signals from slowly moving stimuli reach V1. Indeed, when tested at low speeds (<10° s^−1^), she had a range of visual capacities related to motion (Rizzo et al., 1995) and could reach for objects moving at relatively slow speeds though, even here, her performance was imperfect (Schenk et al., 2000). Her speech reading was much impaired but she could report actions that occurred slowly (Campbell et al., 1997), emphasizing yet again the divide between fast and slow motion. We note that LM’s (imperfect) capacity for detecting slow motion may also be the consequence of activity in other areas besides V1, possibly including area V3 (Shipp et al., 1994).

An attempt to show motion deficits in monkey V5 by injecting µl quantities of the neurotoxin ibotenic acid into it have also led to defects in motion perception, the consequence being a severe elevation in thresholds for motion detection, but not for detection of contrast. The overall evidence from these studies shows that the monkey can recover from such lesions but that further injections of ibotenic acid can also lead to a permanent perceptual deficit, though one that is reflected in an elevation of thresholds for motion detection (Newsome and Paré, 1988), not dis-similar to the elevation in thresholds for wavelength discrimination following V4 lesions in monkeys, briefly described (Fries and Zeki, 1979). Even when permanent, however, the deficits do not begin to match the more severe, global and permanent deficits observed in patient LM. The recovery is interesting, too. Patients whose V1 is damaged, leaving V5 deprived of an input from it or a return output to it, can nevertheless improve their conscious detection of motion significantly after training (Huxlin et al., 2009), implying yet again that neither an input from V1 nor a return output to it are essential for conscious awareness.

#### Activity at Nodes and Awareness

The ability of V5 to sustain a crude but conscious awareness of visual motion, even when disconnected from V1, raises a host of interesting questions about the role of stations along the parallel pathways leading to an area and from it, since it is now generally accepted that each of the parallel visual pathways consists of several stations, or nodes. In the visual motion pathway, the connections between V1 and V5—both the direct ones and the indirect ones through V2—have been well charted and studied (Shipp and Zeki, 1989a,b; Figure 3). Moreover, V5 projects to several other areas, so that it is by no means a terminal node, at least anatomically.

In general terms, it is difficult to suppose that the sole purpose of whatever processing takes place at a given node within a hierarchical pathway is to relay the result of that processing to the next step in the hierarchical chain. This would mean that what is processed at that node would be lost to perception, which would constitute a considerable waste of neuronal resources (Zeki and Bartels, 1999). It is much more likely that something at least of what is processed at a given node becomes available to perception and therefore to awareness, which is not to imply that the result of what is processed at that node is not also passed on to the next node in the hierarchical chain; a node could of course play other roles that may not be directly accessible to consciousness, for example reduce noise, filter information or act as an error detector. Perceptually, the direction of motion of slowly moving stimuli are discernible and could be mediated by activity in V1 or V2 which acquires a conscious correlate. There is no good reason to suppose that it is only activity at a subsequent node that makes them so. Hence, one must suppose that activity at each node can acquire a conscious correlate, even if it is an impoverished one. V5 provides a very good example of this since, through activity in it even when de-afferented from V1, subjects are able to perceive consciously what it processes. This is a general pointer to the results of activity at other stations of other pathways, specialized for other attributes. But it should not be understood to imply that V5 acts alone, without contributions from other nodes or areas in the visual or other systems. Indeed, it is likely that there are enabling systems that are necessary for V5 activity to reach a conscious correlate (Zeki and ffytche, 1998).

#### V5 and Perceptual Asynchrony: A Central Problem for Brain Studies

Given the central role of V5 in motion perception and of other visual areas in the perception of other attributes, it becomes interesting to ask the more general question of how attributes processed in separate areas are combined, if indeed they are, to give us an apparently seamless, unified picture of the visual world. Here, I am concerned with the problem of binding across attributes and not within them, the latter a topic that has been extensively reviewed (Engel et al., 1992, *inter alia*).

It has long been assumed, either implicitly or explicitly, that different attributes of a visual scene, such as its color, form and motion, are processed and perceived simultaneously, an easy assumption to make given our unitary experience of the visual world, where all attributes are apparently seen in perfect unison. But pairing experiments, where subjects are asked to pair the color and direction of motion of a single stimulus, presented briefly and centrally in the field of view, or of two separate stimuli presented in the two visual hemifields—one changing in direction of motion and the other in color—show that we perceive (and thus become aware of) color some 80 ms before we become aware of motion (Moutoussis and Zeki, 1997a; Arnold et al., 2001; Viviani and Aymoz, 2001; Linares and López-Moliner, 2006; Self, 2014). Hence, activitities at given stations in the visual pathways acquire perceptual correlates at different times, in milliseconds. Nor is perceptual asynchrony restricted to motion and color; there is as well an asynchrony in the perception of color and form (orientation) (Moutoussis and Zeki, 1997b). There are conditions where such an asynchrony may not obtain, for example when the pairing is between the change in color and in the direction of motion (temporal order judgments) (Bedell et al., 2003; Clifford et al., 2003), an altogether different kind of pairing. These, though of interest in showing that simultaneous changes, no matter in what attribute, may be simultaneously perceived, are of lesser interest for my argument here. The problem, then, is two-fold—to account for the perceptual asynchrony over very brief time windows on the one hand and for how this asynchrony resolves over longer periods on the other.

Perceptual asynchrony does not address the question of how long it takes to process a stimulus. Rather, it uses an end-point—perception—to measure the *relative* times that it takes to process stimuli to a perceptual awareness. When considered against the demonstration that we become aware of different attributes because of activity in different, functionally specialized, visual areas, the results of temporal asynchrony experiments thus suggest that there are many microconsciousnesses, distributed in space and time (Zeki, 2003). Perceptual asynchrony is also a strong pointer to the massively asynchronous operations of the brain, raising the general question of how binding occurs in such an asynchronous system, including how the micro-consciousnesses, generated separately in space and in time, are bound. Yet, surprisingly, it has attracted relatively little attention.

Perceptual asynchrony is likely due to differences in processing times taken to bring signals related to motion and to color (and other attributes) to a perceptual endpoint. The perceptual asynchrony of motion relative to color can be shortened, by the simple expedient of manipulating the direction of motion that is to be paired with color; such manipulations produce more or less inhibition in V5 cells (Priebe and Lisberger, 2002; Priebe et al., 2002), thus providing good evidence in favor of this supposition (Arnold and Clifford, 2002; Linares and López-Moliner, 2006). Further evidence is provided by pairing motion with motion; whereas the pairing of left-right with up-down motion is synchronous (presumably because of similar degree of excitation and inhibition produced by the two pairs of stimuli), the pairing of up-down motion with motion that is up and to the right results in an asynchrony in favor of up-right motion, presumably because the latter entails less inhibition (Lo and Zeki, 2014). Equally, luminous motion has a temporal advantage over isoluminant motion (Lo and Zeki, 2014), presumably because V5 cells respond more vigorously to luminant than to isoluminant stimuli (Saito et al., 1989; Gegenfurtner et al., 1994; Seidemann et al., 1999). Paradoxically, when pairing across space, the pairing of directions of motion in the two hemifields takes precedence over the pairing of colors, which is probably also attributable to cortical processes and conduction velocities. Fibres connecting V5 of one hemisphere with its counterpart in the opposite hemisphere are heavily myelinated and therefore likely to carry signals faster than the lightly myelinated axons connecting the two V4s (Bartels and Zeki, 2006). This constitutes further evidence of the dependence of perceptual asynchronies on differences in processing times (see Figure 4). Thus, the activity of cells within a single area may reach a perceptual end-point before the activity of other cells in the same area, consistent with notions of “quantized” awareness propounded by Escobar (2013). This implies that asynchronous operations are much more ubiquitous than even the original asynchrony experiments envisaged.

Regardless of whether one is a proponent of hierarchical or parallel processing strategies in the visual brain, these findings create a critical issue for learning how the brain binds attributes processed by different, specialized, systems. They even create a problem for those who believe in neither processing strategy, implicit in what they call “multiplex” cells, that is to say ones that code for all three attributes—color, form, motion (Leventhal et al., 1995; Shapley and Hawken, 2011); they must account for how cells “wait” for different attributes to be processed to completion, that is to reach a perceptual end-point (Zeki, 2015). Perceptual asynchrony experiments, by showing that over very brief time windows subjects mis-bind the two attributes presented simultaneously on the screen (Moutoussis and Zeki, 1997a), suggest that there is no “waiting”. These findings have led us to propose that binding between attributes is post-perceptual (Rangelov and Zeki, 2014).

As interesting is the output related to behavior. The asynchrony experiments, originally derived from pairing color with motion or color with orientation (see above) suggest an asynchronous behavioral output from the color, motion and form systems. When subjects are asked to identify the color and orientation of briefly presented stimuli, the errors made in correctly identifying color and form (orientation) are independent, even under conditions of focal attention (Rangelov and Zeki, 2014), almost certainly due to the fact that color and form (orientation) are processed independently. This would, of course, not be so if color and orientation are “bound” in single cells.

Hence, to summarize, whether the parallel inputs to an area deliver their signals synchronously or asynchronously, all the indications are that the processings undertaken by an area are not necessarily synchronous and that the outputs from them are not necessarily synchronous either, and nor are the top-down inputs to them.

Collectively, these results suggest that the brain is a massively asynchronous organ, with no central clock that resets the activity in each of its parallel systems (Zeki, 2015). The motion system, based on V5, has played a critical role in this. Asynchronous processing is, I believe, something that future experiments and theorizing about the brain must take into account. Parallel, asynchronous, operations make for a more efficient brain because the fastest processing system does not have to “wait” for the slowest one to complete its task. Computer scientists are struggling to develop asynchronous computers, which would be more efficient than the current synchronous ones (Sutherland and Ebergen, 2002). They are, in fact, struggling to make computers that operate more like the brain.

### Conclusion

This review on V5 is evidently done through the prism of my own interests and my own work. There are many excellent papers which I have not referred to, and for this I apologize to all those who may be offended by my omissions. My aim has been to portray, within a limited space, work on V5 which has seemed to me to be especially illuminating in clarifying how it operates and in giving hints about how other visual areas may operate as well.

From all that I have written above about V5, it can be concluded that there are strong and major dividing lines, at different levels, which constitute a sort of conceptual framework for thinking about V5. These are between: cortical and direct subcortical inputs to it, between fast and slow motion, between hierarchical and parallel processing, between activity of separate groups within V5 that process separate characteristics of motion separately and those that process them jointly, between synchronous and asynchronous processing and between cells whose activity acquires a conscious correlate and those that do not. How cells that fall on either side of these divides collaborate to sculpt the overall physiology of V5 and determine its overall role in visual perception is an exciting problem for the future. Resolution of these problems may in fact give strong hints about how other visual areas, with other visual specializations, resolve similar, if not identical, problems. In the foreseeable future, V5 is very likely to continue giving important insights into how the visual brain operates, as it has consistently done in the past.

V5 has truly been, and continues to be, a microcosm of the visual brain.

## Conflict of Interest Statement

The Guest Associate Editor Sharon Gilaie-Dotan declares that, despite being affiliated to the same institution as the author Semir Zeki, the review process was handled objectively and no conflict of interest exists. The author declares that the research was conducted in the absence of any commercial or financial relationships that could be construed as a potential conflict of interest.
